# Variation in trends of consumption based carbon accounts

**DOI:** 10.1038/s41597-019-0102-x

**Published:** 2019-06-24

**Authors:** Richard Wood, Daniel D. Moran, João F. D. Rodrigues, Konstantin Stadler

**Affiliations:** 1 Industrial Ecology Programme, Norwegian University of Science and Technology (NTNU), Trondheim, Norway; 2 Institute of Environmental Sciences CML, Leiden University, Einsteinweg 2, 2333 CC Leiden, Netherlands

**Keywords:** Climate-change policy, Sustainability

## Abstract

The UNFCCC requires the annual reporting of greenhouse gas emissions. These inventories focus on emissions within a territory, and do not capture the effect of emissions embodied in imports. Consumption based carbon accounting (CBCA) has been proposed as a complementary method to capture these emissions, and a number of global models have been developed to operationalise CBCA. However, discrepancies in country-level CBCA results occur, which can cause concern for the practical use of CBCA. Despite these quantitative difference in results, do they provide robust results when changes over time are investigated? Here we present results of all the major global models and normalise the model results by looking at changes over time relative to a common base year value. We give an analysis of the variability across the models, both before and after normalisation in order to give insights into variance at national and regional level. A dataset of harmonised results (based on means) and measures of dispersion is presented, providing a baseline dataset for CBCA validation and analysis.

## Introduction

The United Nations Framework Convention on Climate Change (UNFCCC) requires the annual reporting of territorial greenhouse gas emissions from Annex 1 countries^1^ (which equates to “production based carbon accounting” (PBCA) with specific handling of international transport fuels such as bunkers). There has been discussion about whether PBCA of emissions alone are adequate, as the evidence grows that the de-carbonisation of some developed countries was at least partially due to the growth of energy intensive industries in developing countries^2,3^.

Consumption based carbon accounting (CBCA) has been proposed as a complementary form of accounting. CBCA (also called carbon footprints) capture the emissions that occur globally for the actual goods and services consumed in a country^4^. They are calculated based on the “embodied” emissions that are associated with the production, transport and sale of a good.

Recent advances in global multi-regional input-output (MRIO) modelling has led to the development of a number of large databases suitable for calculating consumption based carbon accounts^5^. Due to construction challenges in MRIO databases, incomplete data, resource constraints, and underdeveloped accounting standards, estimation steps are required and accounting practices are not uniform. Furthermore, MRIO databases are often constructed with slightly different aims, for example to focus on trade, or individual accuracy at the country level. This leads to a degree of inconsistency among MRIO databases^6^. While CBCA are often perceived as less robust than emission inventories, several studies have shown that at the national level the accuracy of CBCA results is not significantly different than PBCA^7–9^. However, there are still a huge range of results being presented as carbon footprint results. In order to set policy targets using carbon footprint values there is a clear need for a harmonized dataset and an indication of reliability of footprint accounts.

The main source of difference between CBCA results from different models typically stems from the domestic emission inventory linked to the economic IO model^10^. The estimated volume and composition of consumption (also called final demand) is another cause of variation between models^11^. The tracking of embodied emissions through global supply chains (i.e. trade and transformation steps) is, surprisingly, relatively robust across models^11^. The trade results do differ between CBCA, but this is not the main cause of divergent results^6,12,13^. In general, results from different MRIO databases show significantly more agreement at the country level than the product level^14^.

Comparison work and evaluation of robustness to date, however, has somewhat overlooked the main use of CBCA data – the tracking of whether (and by how much) emission accounts are increasing or decreasing over time, and whether these changes are similar or different to changes in the territorial account. By analyzing the time trend it can indicate progress toward targets and evaluate whether there has been a growth or decline in emission transfers. At least for the original Kyoto targets under the UNFCCC, they were principally based upon greenhouse gas emissions relative to a 1990 baseline. As emission estimates get retroactively revised as better emissions accounts become available, the importance lies upon having consistency in the data used to estimate change over time.

The purpose of this work is to report the trends in time of country-level PBCA and CBCA, as reported by different models, and to explore how these trends are affected when absolute emissions are normalized to a baseline year (2005) and the original yearly growth rates are preserved. We report the dataset of PBCA and CBCA values, including mean values (“harmonised results”) and measures of dispersion. The work is aimed to complement and simplify the interpretation of robustness of top-level CBCA results and not at replacing individual MRIO databases and associated analysis, which are often designed with a specific focus.

We collect results from five available MRIO models: EXIOBASE (49 regions, high product detail, 1995–2016), WIOD (41 regions, low product detail, 1995–2009), Eora (187 regions, variable product detail, 1970–2015), the ICIO (61 countries, 34 industries, 1995–2011), and the use of GTAP through the results presented in the Global Carbon Project (130 regions, 1990–2016). These five databases are described in more detail in the methods (section 4.1). Due to the fact we have only a small number of “observations” in the form of model results that could expect to have correlation, we report both relative standard deviation and relative mean absolute differences (see methods) in the tabular results, and care must be taken in the interpretation here (they provide a measure of model variability and not of uncertainty of a CBCA account).

In section 2 we present key results in a graphical format, with a short discussion in section 3. In section 4, we outline the methods used to normalize the PBCA and CBCA. The dataset of PBCA and CBCA emissions and their variation is attached to this article as a supplementary MS-Excel file and is also available in the scientific data repository Zenodo^15^, alongside code^16^.

## Results

### Regional trends

**Fig. 1:**
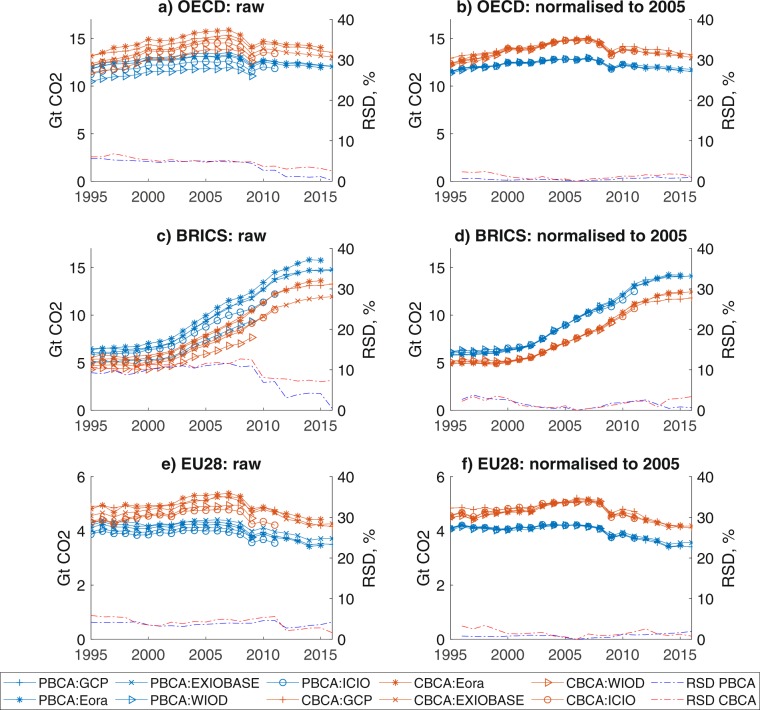
PBCA and CBCA results for major regions calculated from each of the five models, with relative standard deviation (right axis). (**a**) OECD countries raw model results; (**b**) OECD countries after normalising to 2005; (**c**) BRICS raw model results; (**d**) BRICS after normalising to 2005; (**e**) EU28 raw model results; (**f**) EU28 after normalising to 2005.

In this section, we present the *raw* model results and results *normalised* to a common base year of 2005, as well as a measure of their variation. Raw results are results taken directly from the original models, whilst normalised results look at the year on year change from a common value per country taken as the mean of model results for 2005 (see methods). Variation in headline results for raw model results of large regions are in the order of 5–10% using a measure of relative standard deviation (RSD) (Fig. 1, see dataset for all country results). Normalising the production and consumption results to a common base year (2005) reduces the relative standard deviation to in general below 5%. Due to the nature of the normalization (and choice of base year), RSD measures are zero for the base year (2005) of the normalized results, and in general, increase the further away from the base year. A sensitivity analysis was performed on different base years, but the overall results were similar – at least a halving of RSD when comparing raw and normalized results.

### Country level variations

Whilst most major economies and regions have relative standard deviations across the 5 models in the order of 5–10% for both PBCA and CBCA results before normalising, and circa 1–2% after normalising, this does not necessarily hold for individual countries. Countries that are highly trade exposed, or with large international transport emissions (e.g. shipping of Greece) show the highest amount of variability due to the different conceptual coverage used in different MRIO models (for example, residential or territorial principle^17^). For such economies, raw result variability is in the order of 20–30%, and is roughly halved after normalising (Table 1). In Table 1, the average measure of variability (over time) is reported both to the raw and normalised data. Both relative standard deviation (RSD) and relative mean absolute differences (RMD) are reported (see methods). Overall we see that normalised values show better agreement than raw values for almost all results, and that production accounts show more convergence than consumption accounts. The RMD (capturing the mean of dispersion between pair-wise model results) values are smaller than the RSD values (dispersion relative to the mean). The larger variation in the CBCA accounts than the production accounts is expected due to the difference in economic data used to do the allocation between production and consumption between the different MRIO models. Small trade-exposed countries such as Hong Kong, Singapore and Luxembourg show the highest variation. Here variation in CBCA results are often significantly higher than in PBCA results, implying that handling of international trade in the transaction accounts in the MRIO models differ (compared to handling of emissions of international transport, which would also show similar variation in the PBCA results, see e.g. Greece, Denmark). In general, care should be taken in looking at results for such countries (regardless of PBCA or CBCA).

**Table 1:** Variability of PBCA results and CBCA results between models, by country

Region	PBCA RSD raw	CBCA RSD raw	PBCA RSD norm	CBCA RSD norm	PBCA RMD raw	CBCA RMD raw	PBCA RMD norm	CBCA RMD norm
OECD	4.2	5.0	0.5	1.2	4.0	4.9	0.5	1.1
BRICS	9.0	10.4	1.6	1.7	8.4	10.1	1.5	1.6
Annex I	6.0	6.7	0.4	1.0	5.8	6.5	0.4	1.0
G8	3.5	4.3	0.4	1.0	3.4	4.2	0.4	1.0
EU28	3.8	4.4	0.8	1.6	3.7	4.3	0.8	1.5
Global	4.9	5.5	0.9	0.9	4.8	5.4	0.8	0.8
Australia	4.3	7.4	1.9	4.1	4.1	6.4	1.7	3.8
Austria	8.0	6.6	1.6	2.3	7.8	6.3	1.5	2.2
Canada	5.3	7.9	1.6	4.9	4.9	7.6	1.5	4.6
Chile	2.9	3.7	1.6	3.7	2.4	3.1	1.4	3.1
Denmark	19.3	8.8	8.3	4.2	17.2	8.1	7.2	4.0
France	4.4	6.1	1.0	2.7	4.1	5.7	0.9	2.5
Germany	4.9	5.5	1.4	2.7	4.7	5.1	1.3	2.6
Greece	33.6	23.8	3.2	8.9	27.5	22.9	2.8	7.9
Italy	3.4	5.1	1.6	3.1	3.2	4.9	1.4	2.9
Japan	4.1	8.7	0.9	2.0	3.8	8.3	0.9	1.8
South Korea	4.2	10.5	2.6	5.7	4.0	9.8	2.3	5.0
Luxembourg	20.3	41.1	10.8	17.5	17.2	36.6	8.7	15.3
Mexico	6.7	6.0	3.1	2.9	6.5	5.8	2.8	2.7
Netherlands	4.6	9.4	1.3	6.2	4.3	8.9	1.3	5.9
Norway	22.7	19.8	2.4	9.2	19.9	18.0	2.3	8.4
Portugal	4.8	9.4	3.4	5.2	4.7	8.7	3.0	4.6
Spain	5.1	10.3	1.3	2.7	4.8	8.8	1.2	2.5
Sweden	5.1	8.5	3.8	3.1	4.8	7.9	3.6	2.9
Switzerland	10.3	11.8	3.2	5.6	8.8	10.8	2.6	5.1
Turkey	6.8	10.5	2.1	2.9	6.5	9.9	1.9	2.8
U.K.	4.9	6.1	1.7	3.2	4.6	5.8	1.6	2.9
U.S.A.	4.2	3.7	0.6	1.1	3.9	3.5	0.5	1.0
Argentina	2.4	5.1	1.6	3.9	2.0	4.2	1.3	3.3
Brazil	8.7	8.9	3.4	2.3	8.2	8.6	3.0	2.1
China	8.0	9.2	2.8	3.7	7.6	8.8	2.6	3.5
Hong Kong	2.4	71.6	1.0	17.4	1.7	50.6	0.7	12.3
Indonesia	7.7	8.8	5.4	4.4	7.0	8.1	4.5	3.9
India	5.3	5.8	1.6	2.6	5.0	5.6	1.5	2.5
Russia	6.4	13.9	1.4	6.2	6.0	13.6	1.2	5.9
Singapore	13.6	40.8	20.8	18.0	11.2	34.0	16.9	14.8
Thailand	7.7	11.9	2.3	8.6	6.4	10.2	1.9	7.1
South Africa	11.7	12.0	4.8	8.6	10.7	10.7	4.4	7.9
Average	8.3	11.9	3.6	6.5	7.3	10.7	3.1	5.8

RSD is relative standard deviation, RMD is relative mean absolute deviation. Values show the numeric average across all years. “raw” means original MRIO results; “norm” means normalised to the 2005 base year. Additional countries available in supporting information.

The naïve (unweighted across all regions) average of the relative standard deviation (RSD) over all model observations is 8% for the PBCA, and 12% for the CBCA. This is prior to the normalising of the emission accounts. After normalising to a common base year the naïve average relative standard deviation drops to 4% for the production account and 7% for the consumption account. The lower variation for normalised compared to raw results means that time series trends across models show less variation than the absolute values. Thus when investigating whether there is an increase (or decrease) in the displacement of emissions due to trade, the normalised results in general show good levels of reliability.

### Relation between variation and magnitude of emission estimates

One question that often confronts analysts is whether the robustness of results increases for larger countries. In theory, emission accounts for a larger country are made up of many more observations than those for a small country, and are generally supposed to be better known. In order to investigate whether this actually occurs, we investigate the relationship between scale and variance for both raw and normalised data and PBCA and CBCA.

**Fig. 2:**
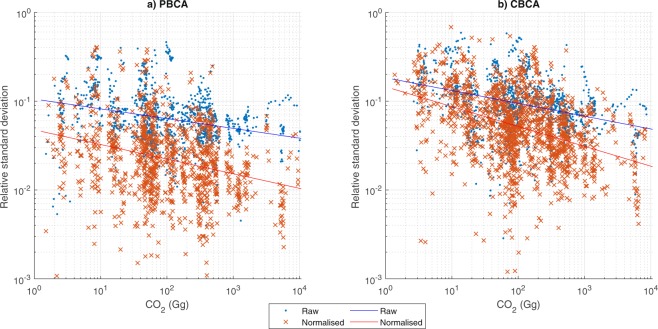
Relationship between relative standard deviation and mean emission values plotted for 61 countries and when more than 3 model observations for the period 1990–2016. LogLog figure, regression lines plotted based on least squared linear regressions of the log-transformed data. (**a**) Results for PBCA. (**b**) Results for CBCA.

Figure 2 provides a complete plot of all observations of raw data for both production and consumption accounts (i.e. one data point per country per year, where there are at least 3 different models reporting values), along with linear least-squares (LSQ) curve fitting of the log transformed accounts. Log transformation was done due to the skewed number of results for large versus small values. Linear least-squares curve fitting was applied to give a simple indication about whether there was higher variability for large vs small accounts (see discussion in literature^8,14^). In general we find that there is more variation among models concerning CBCA rather than PBCA (as the linear regression of the RSD values of (log-transformed) CBCA is greater than that of the corresponding PBCA data). This general pattern is, however, not very strong, since the difference in RSD between regression lines is around 1% in the whole range of absolute emissions (compare the blue lines between the left PBCA and right CBCA plot). Another noticeable pattern is that the regression line is downward sloping, meaning that on average larger economies exhibit less variation in the estimates of PBCA and CBCA across models. Interestingly, we obtain much higher levels of convergence for large economies after normalising to 2005 (normalized results have a larger negative coefficient for both PBCA and CBCA than the raw results).

## Discussion

This work provides an emissions dataset of production and consumption based carbon accounting (PBCA and CBCA) results as well as an assessment of robustness of these results, both with, and without normalising results to a common base year. Results are presented at the national level for both production and consumption perspectives. All major economies available across the MRIO databases were included, as well as regional aggregates. We see that the main source of inter-model variation drops from a relatively high level for raw MRIO results (between 5–10% for major regions using relative standard deviation as a measure of precision, up to 30–40% for individual countries) to a relative low level after normalizing (1–3% for major regions, and up to 20% for outlier countries). The dataset is intended as a ready to use dataset for country and regional PBCA and CBCA results. The normalised mean for each country/region is the principle result that can be used to assess the magnitude and trend in the emission accounts. However, an additional key element of the dataset are the measures of robustness and spread of the results across the source models. These metrics give insight into the amount of trust that should be placed in the individual country/region results. Example uses of the dataset are for determining whether CBCA results are trending in line with PBCA results, or whether there is a growth in the gap between PBCA and CBCA results which shows increased emissions embodied in trade.

This work focuses on interpreting top-level trends over time, and is complemented by recent work that has aimed to understand why MRIO databases and associated results vary, taking a cross-sectional perspective (see work, in particular, by Owen^18^). Whilst the implementations of each MRIO database are performed independently, projects do share many source data (UN System of National Accounts totals, COMTRADE data, country level supply and use tables etc.) and similar approaches. A range of work has sought to develop techniques for evaluating how these implementations manifest themselves into different results, often at the supply-chain level^9,10,12,13,19,20^. The choice of raw emission data as well as the method of handling energy consumption in the economy are highlighted as the factors that have the major effect on the difference between MRIO model results^10^. Other authors^21–23^ have investigated the effects of sectoral and country aggregation. This type of work can help MRIO builders in particular to understand the differences that certain data choices can have in the realization of final results, and also where to focus additional efforts in country or sector disaggregation. Monte-Carlo assessments have also been performed^7,8,11^ to give insight into the expected level of stochastic uncertainty (advanced by Rodrigues *et al*.^14^ by including the effect of covariance between model elements). In general, the work has found that in descending order of priority, the emission account total, the emission account sector-wise allocation, the final demand total and composition, and lastly the structure of the economy (the technical coefficients matrix) were the largest drivers causing variation across databases^10–12^. In comparison to the work here, we find quite similar results when looking at the cross-sectional results at a certain point of time – the fact that most PBCA and CBCA vary in a similar amount of magnitude point to the need for benchmarking to a consistent emissions account. Interestingly, the stochastic work on uncertainty often reports expected relative standard deviations to a similar order of magnitude as to the inter-model comparison here.

Some of the papers discussed above also investigate the relationship between the size of CBCA estimates and their relative uncertainty. The basic premise is that large values are relatively easy to measure (or based on an aggregate of more independent data points), and thus have lower values of uncertainty compared to small values. This, for example, was found in the analysis of raw uncertainty data used to construct an early UK-MRIO^8^ and in the data from the GTAP database^7^. Recently, Rodrigues *et al*.^14^ in their uncertainty analysis of the same five models as here (and accounting for data correlations) did not find evidence to support these relationships. Different approaches are being taken (uncertainty analysis of raw data, vs a simple assessment of agreement in final results). However, we clearly find higher levels of agreement in model outcomes for larger regions than smaller regions. Further analysis could look at whether the level of trade-exposure and significant presence of international transport in the region affects results. A basic overview of our results does show some results to support such a hypothesis (Table 1), albeit the handling of international emissions from bunkers has probably the greatest effect here. For further details we refer to in depth analysis of this issue in the literature^12,20^.

Previous work on uncertainty has also indicated that the errors associated with CBCA are basically the same as PBCA. Whilst clearly in the same order of magnitude, we do find a systematic higher level of variation in CBCA in comparison to PBCA (Fig. 2), both for *raw* accounts and for the *normalized* accounts. Normalisation (as mentioned earlier) reduced the expected variation of the CBCA significantly. However, normalized results show CBCA to have a RSD of roughly twice that of the PBCA (Fig. 2). Whilst no literature is directly comparable, this contradicts earlier findings in Peters *et al*.^20^, who found lower levels of disagreement between CBCA than PBCA, but supports the general findings in Karstensen *et al*.^7^ and Moran and Wood^11^.

In our work, we took a reference year of 2005 – the mid-point of the time-series. The choice of reference year will affect results, with measures of variance being higher the further distant from the reference year. Two other choices were tested in our modelling (and the accompanying code allows for easy replication with any choice of reference year), 1995 and 2009 – the first and last years when all model results were available. The differences were not substantial, and do not affect the qualitative conclusions of the work.

Finally, the data set here is intended to help make accessible results on top level findings from the MRIO/CBCA research field, along with an assessment of robustness on those results. It helps offers comparison results that can help support policy decisions, and confirms the degree to which different models report similar trends. The results clearly point to a high level of convergence for most major regions around the world when trends over time are investigated. For those countries where CBCA results are still heading in a different direction to PBCA results, the data set can provide further motivation to improve official reporting of such results.

## Methods

### Data sources

We collected five of the current leading MRIO models that provide CBCA carbon footprint results. We focus on CO_2_ emissions from fossil fuel combustion, although the Global Carbon Project (based on GTAP, see below) inventory also includes emissions from cement production. Emissions from cement production are ~2% of global CO_2_ emissions but can be a larger fraction in some cases, e.g. they have been estimated to account for up to 5% of total emissions in China (for a discussion see Andrew^24^).

We compare results at the national level. The MRIOs have different degrees of sectoral detail, ranging from none (Global Carbon Project) to a heterogeneous level of detail per country (Eora), to consistent high resolution for a limited set of countries (EXIOBASE), so this is not considered in this work. There are a no other global multi-regional models currently available, albeit IDE-JETRO^25^ provides an Asia focused model, and the Global IELab^26^ is being set up to flexibly generate future MRIO models, but does not yet have results.

Here we summarize the five MRIOs that are included:

**EXIOBASE**. The EXIOBASE database covers 44 countries and 5 rest of world regions, and in each region has 163 industries and 200 product categories. It includes sector-specific data for gross energy use, emission relevant energy use, and gross energy supply for around 60 energy products, and has air emission data for 27 types of air emissions of both combustion and non-combustion emissions. It covers the years 1995 to 2011, as a basis, but has been extended to 2016^27,28^. The energy and emission accounts in EXIOBASE follow Eurostat’s guidelines^29^ and the UN SEEA accounting standards^30^. The energy accounts are based on data from IEA’s energy balances (after converting to residential principle) and emissions related to combustion were calculated by multiplying emission relevant energy use data with emission factors obtained from the TNO Emission Assessment Model^31^. The latest version at time of writing (v3.7) was used in this work. EXIOBASE is available from www.exiobase.eu, and the version used here is archived at https://ntnu.app.box.com/folder/50207742482, and available from the authors on request.

**ICIO**. The inter-country input-output (ICIO) table published by OECD is a symmetric input-output table covering 61 countries and the rest of the world, with 34 sectors of detail, for the years 1995–2011^32^. ICIO’s main data sources are OECD and UN SNA data, national IO/SUTs, OECD COICOP consumption data, OECD tourism satellite accounts, UN Comtrade and OECD ITCS and trade in services estimates. ICIO uses the IEA CO_2_ from fuel combustion data to estimate the CO_2_ emissions by industries. The IEA fuel combustion data is based on IEA energy balances^33^ combined with the IPCC Guideline emission factors^1^. Downloaded from oecd.org. 2016 Edition.

**Eora**. Eora is a global MRIO database with 187 individual countries. It uses variable sectoral resolution from 26 to ~500 sectors for a total of 15909 sectors. Using heterogeneous classifications in each country allows the system to preserve national IO table detail wherever possible. Eora covers the period 1970–2015. The monetary MRIO tables are complemented with satellite accounts containing physical data at a detail of 35 indicator groups^34,35^. The Eora database uses a constrained optimization approach to construct an emissions inventory. The primary data source is the EU JRC Emissions Database for Global Atmospheric Research (EDGAR) database of GHG emissions, but national and international data are also used as constraints where available. The emission inventory is not converted into the residence principle. The emissions are first allocated to sector groups according to emissions category, then within those groups allocated to subindustries according to industry output. Downloaded from worldmrio.com. Version 199.82.

**WIOD**. The World Input-Output Database (WIOD) covers 40 countries and a single rest of the world region. It uses a homogenous sector classification with 35 industries in each country^36^. WIOD is built by combining information from national statistic institutes and UN COMTRADE. The WIOD database covers the period 1995–2011. WIOD’s environmental accounts include data on energy, air emissions, materials extraction, land and water use, but cover only the period 1995–2009^37^. The energy and emissions data is presented according to the residence principle and the preferred source of data is from National Accounting Matrices including Environmental Accounts. When that is not available, inventories are constructed based on the energy-first approach, and by applying emission factors. Downloaded from wiod.org. Release 2013. It can be noted that in some results WIOD commonly provides lower estimates for the large regions than other models. WIOD is based on older estimates of energy use and emissions than the other MRIO models, which have had more recent updates.

**GCP**. Global Carbon Project provides production and territorial emissions accounts for 120 countries. For production-based accounts the database covers the period 1959–2017. For consumption-based accounts the database covers the period 1990–2016^38^. GCP CBCA is built starting from the GTAP8 database^39^, which has a resolution of 140 countries and 57 sectors, and then extrapolated to estimate an emissions time series dataset^20^. The construction process for this is described in detail by Peters *et al*.^2^. GCP benchmarks its emissions data to the CDIAC and EDGAR emissions databases to interpolate an emissions timeseries. Downloaded from 10.18160/GCP-2018 on January 2019.

### Emission calculations

Eora, and GCP provide PBCA and CBCA results per country directly. For EXIOBASE, ICIO, and WIOD, CBCA results were calculated using the standard Leontief demand model^40,41^ as below.

For the raw model results, the production account (emissions by country of origin) of each MRIO database *m*, year *t*, and for each country *r*, ${PBCA}_{m,r,t}^{raw}$ can be represented as the sum of sectoral, *j*, CO_2_ emissions **F**_*m,t*_ plus the direct household CO_2_ emissions per country ${f}_{m,t,r}^{y}$ where for ease of manipulation, emissions of each country *r* in **F**_*m,t*_ and ${{\bf{f}}}_{{\boldsymbol{m}},{\boldsymbol{t}}}^{y}$ are represented on separate rows so ${PBCA}_{m,t,r}^{raw}={\sum }_{j}{F}_{m,t,r,j}+{f}_{m,t,r}^{y}$.

The consumption account of the ${CBCA}_{m,r,t}^{raw}$ represents emissions attributed to final demand of a country. The standard Leontief demand model is used to attribute sectoral emissions to final demand:

$${CBCA}_{m,r,t}^{raw}={{\bf{s}}}_{m,t}{({\bf{I}}-{{\bf{A}}}_{m,t})}^{-1}{{\bf{y}}}_{m,t,r}+{f}_{m,t,r}^{y}$$

where **A**_*m,t*_ is the inter-industry matrix for each MRIO model, and **s**_*m,t*_ is the sector specific emissions $\left({\sum }_{r}{F}_{m,t,r,j}\right)$ per unit output, and **y**_*m,t,r*_ is the final demand of dimension row *j*, for each country *r*. PBCA and CBCA for each model are pre-calculated in this work, and can be performed using pyMRIO^42^.

Emission calculations are performed across a set of 61 countries and 10 regions (see appendix), with results stretching back to 1960 and forwards to 2016. Aggregation to regional results is performed subsequent to the calculation of country level results. Whilst in general, the MRIO models provide the necessary detail for the country level results, some databases do not provide the necessary resolution for regional results (e.g. EXIOBASE misses Argentina and Saudi Arabia from the G20). In this case, the results are scaled by contribution to GDP.

### Normalising to a common base year

We firstly define a growth rate variable ${CBCA}_{m,r,t}^{gr}$, that shows the changes in the CBCA from year *t−1* to year *t*:

$${CBCA}_{m,r,t}^{gr}=\frac{{CBCA}_{m,r,t}^{raw}}{{CBCA}_{m,r,t-1}^{raw}}$$

We then choose a common base year for model results, taking a central year of all model results (2005). We also tested and scripted the code to work with any choice of base year between 1995–2009, with minimal impact on quantitative results. We define the mean of the CBCA of all MRIO database results for region *r* as

$${\bar{CBCA}}_{r,t=2005}=\frac{{\sum }_{m}{CBCA}_{m,r,t=2005}^{raw}}{{m}_{obs}},$$

where *m*_*obs*_ is the number of model observations for each country in 2005 (noting that not all models report values for every country). Assigning the value of the mean to all database results for the base year, gives us the starting value for re-constructing the subsequent normalised time-series:

$${CBCA}_{m,r,2005}^{norm}={\bar{CBCA}}_{r,t=2005}$$

Re-constructing the time-series is then based on growth rates from the base year. Hence for normalising since 2005 (*t* > 2005),

$$CBC{A}_{m,r,t}^{norm}=CBC{A}_{m,r,t}^{gr}\ast CBC{A}_{m,r,t-1}^{norm}$$

And prior to 2005 (*t* < 2005):

$${CBCA}_{m,r,t}^{norm}=\frac{1}{CBC{A}_{m,r,t}^{gr}}\ast CBC{A}_{m,r,t+1}^{norm}$$

The “harmonized” model result is then defined as the mean of the normalised results from all MRIO databases:

$$CBC{A}_{r,t}^{harm}=\frac{{\sum }_{m}CBC{A}_{m,r,t}^{norm}}{{m}_{obs}}$$

The same derivation is repeated for the production account PBCA.

### Measures of variations

No real measure of uncertainty can be calculated because of the small number of observations (number of MRIO databases available) as well as the high levels of interdependence between the databases (sharing many sources of common source data). However, we apply basic statistical measures in order to still capture the amount of variability between the model results and give insight into how this improves by normalising results to a common base year. Because of the small number of model observations, both relative standard deviation and relative mean absolute differences are calculated. The mean is also calculated, in contrast to, for example, the median, based on the general lack of bias or outlier values between models. The mean also gives a reference value for the standard deviation measure. The standard deviation is calculated from the sample (for most regions and years number of observations = 5), and the relative standard deviation is the standard deviation divided by mean of the sample. The relative standard deviation thus gives a measure of variance relative to the mean expected value from our sample. The relative mean absolute difference was also calculated as a separate measure, showing the average distance of each observation from each other (mean absolute difference), relative to the mean. As such, the relative mean absolute difference gives more insight into dispersion between model outcomes whilst the relative standard deviation gives insight into variance relative to the mean of the sample. Number of observations are also reported in the accompanying database.

The formula for the relative standard deviation $RSD(CBC{A}_{r,t}^{norm})$ of any particular account (here for normalised CBCA) follows:

$$RSD(CBC{A}_{r,t}^{norm})=\frac{\sqrt{\frac{{\sum }_{m}{(CBC{A}_{m,r,t}^{norm}-\bar{CBC{A}_{m,r,t}^{norm}})}^{2}}{{m}_{obs}}}}{\bar{CBC{A}_{m,r,t}^{norm}}}$$

The formula for the relative mean absolute difference $RMD(CBC{A}_{r,t}^{norm})$ of any particular account (here for normalised CBCA) follows:

$$RMD(CBC{A}_{r,t}^{norm})=\frac{\frac{{\sum }_{m1}{\sum }_{m2}(CBC{A}_{m1,r,t}^{norm}-CBC{A}_{m2,r,t}^{norm})}{{m}_{obs}^{2}}}{\bar{CBC{A}_{m,r,t}^{norm}}}$$

The calculation of the mean, RSD and RMD, as well as the reporting of number of model observations *m*_*obs*_ was applied to the PBCA and CBCA accounts for the raw model results, the year on year growth rates (e.g. ${CBCA}_{m,r,t}^{gr}$ above) and the normalized model results. Note that the mean of the normalized result is presented as the harmonized result across all model observations as described above.

### Regressions

As is often the case with country level footprints, the distribution of the data is highly skewed. This is even more pronounced when examining absolute and not per-capita footprints. There are many more observations of low orders of magnitude (e.g. footprints of small absolute emitters such as most European countries vs footprints of large absolute emitters such as USA, China). Hence a log-log transformation was applied to obtain a fairly well normally distributed sample. A linear regression was performed over the log-transformed data (i.e. equivalent to a power regression on non-transformed data) in order to investigate the question whether there was higher levels of variability between reports for large observations than small observations (see main text). Other functional forms could be applied, and a number were tested (quadratic, exponential, etc) but none reported better fits measured through r-squared values. Standard Matlab functions were used, which are based on measurements of least-squares. In formulas, where $x=\bar{CBC{A}_{r,t}^{harm}}$ (the expected value, for the case shown here, the mean of the normalised CBCA result) and $y=RSD(CBC{A}_{r,t}^{norm})$ (the corresponding relative standard deviation) regressions were performed to determine coefficients *a* and *b* as:

$$y={bx}^{a}$$

which is equivalent to linear regression when taking logarithms:

$${log}_{10}y=a\ast {log}_{10}x+b$$

## Data Availability

The datasets generated during the current study are available in the *Zenodo* repository 10.5281/zenodo.1296201^15^.

## Code Availability

Analysis code is available on github (https://github.com/rich-wood/CBCA) and on Zenodo^16^.
